# Mr. Mayo on Sir Charles Bell's Discoveries

**Published:** 1834-07-01

**Authors:** Herbert Mayo


*To the Editor of the Medical Quarterly Review.*

Sir: So much effrontery has been of late so systematically shown, in claiming for Sir Charles Bell, at my expense, credit to which he is not entitled, that I am driven to request you will oblige me by inserting the following statement upon the authorship of the modern discoveries respecting the uses of the nerves.

I am, sir, your obedient servant,

Herbert Mayo.

Some time before 1812, Sir Charles Bell printed and distributed an Essay on a New Idea of the Brain and Nerves: a copy of this essay is in my possession. In this essay he describes experiments upon the spinal nerves, which, combined with anatomical observations, led him to adopt the following opinions:

That the anterior roots of the spinal nerves, the anterior columns of the spinal marrow, and the cerebrum, are for sense, volition, and the other affections of consciousness.

That the posterior roots of the spinal nerves, the posterior columns of the spinal marrow, and the cerebellum, regulate growth, nutrition, the sympathies of parts, and the like.

In the Philosophical Transactions for 1821, a paper, by Sir Charles Bell, was published, being the first of a series, on the Uses of the Nerves. In this paper Sir Charles Bell asserted the existence of a set of *superadded* or *respiratory* nerves, which he described as different from the nerves of sense and deliberate voluntary motion, and as channels for conveying the stimulus to muscular action in breathing, and bodily expression. In the face, he said, that the infraorbital and mental branches of the fifth were for sensation and deliberate volition, and that the branches of the seventh formed the superadded or respiratory nerves.

An account of these experiments was published by Magendie, in his Journal of Experimental Physiology, for October, 1821, in which he adopted Sir Charles Bell's views as to a supposed respiratory system; although one experiment upon one branch of the fifth nerve did not, upon its repetition, appear to him satisfactory. Magendie's words are these:

"Nous avons répété ces expériences à l'école vétérinaire d'Alfort, avec MM. Shaw et Dupuy; et le résultat que nous avons obtenu *s'accorde parfaitement avec celui que nous venons de rapporter*, à l'exception toutefois de l'influence de la section *du sous orbitaire* sur la mastication, influence qui n'a pas été évidente pour moi."

It thus appears that the examination of Sir Charles Bell's theory left Magendie in no doubt as to the use of the seventh nerve being what Sir Charles Bell asserted.

In August, 1822, I published, in the first part of my Anatomical and Physiological Commentaries,* a refutation of Sir Charles Bell's theory, that there exists a distinct set of superadded or respiratory nerves. By varied experiments, I proved that the facial branches of the fifth are nerves of sensation alone, and that those of the seventh are nerves of voluntary as well as of instinctive motion. I further observed, that other branches of the fifth, those, namely, distributed to the temporal, masseter, and pterygoïds, must be voluntary nerves.

In following out the latter idea, I came upon the fact, and its physiological bearing, that the ganglionless portion of the fifth is distributed to the muscles just named. I was thence led to conjecture analogically the existence of the true difference of function between the ganglionic and ganglionless roots of the spinal nerves; and I engaged myself in making experiments to establish the justness of my conjecture.

Sir Charles Bell now likewise came upon the same idea. In his first paper in the Philosophical Transactions, it is indeed demonstrably clear that he had not at that time the smallest idea of this difference; and that his views upon the subject had not improved since his early experiments, published before 1812. But now, I repeat, Sir Charles Bell and myself were both intent upon finding evidence to prove the conjectured uses of the double roots of the spinal nerves.

Before, however, either of us had succeeded in proving the fact, Magendie, by ingeniously using very young animals in his experiments, succeeded in obtaining a positive result, and in realizing the discovery, which is honestly his.

To Sir Charles Bell's various publications, in which he claims or assumes credit for discoveries to which he is not entitled, the following words of Seneca would form an excellent motto:

"ISTA PRO INGENIO FINGUNTUR, NON EX SCIENTIÆ VI."

*King's College; June* 3, 1834.

* I beg to refer the reader to this work, and to my "Outlines of Human Physiology," Third Edition, 1833.

